# Non-Invasive Renal Perfusion Imaging Using Arterial Spin Labeling MRI: Challenges and Opportunities

**DOI:** 10.3390/diagnostics8010002

**Published:** 2018-01-05

**Authors:** Fabio Nery, Isky Gordon, David L. Thomas

**Affiliations:** 1 Developmental Imaging & Biophysics Section, University College London Great Ormond Street Institute of Child Health, London WC1N 1EH, UK; i.gordon@ucl.ac.uk; 2 Department of Brain Repair and Rehabilitation, University College London Institute of Neurology, London WC1N 3BG, UK; d.thomas@ucl.ac.uk; 3 Leonard Wolfson Experimental Neurology Centre, University College London Institute of Neurology, London WC1N 3BG, UK

**Keywords:** magnetic resonance imaging, arterial spin labeling, renal MRI, renal ASL, chronic kidney disease, kidney perfusion

## Abstract

Tissue perfusion allows for delivery of oxygen and nutrients to tissues, and in the kidneys is also a key determinant of glomerular filtration. Quantification of regional renal perfusion provides a potential window into renal (patho) physiology. However, non-invasive, practical, and robust methods to measure renal perfusion remain elusive, particularly in the clinic. Arterial spin labeling (ASL), a magnetic resonance imaging (MRI) technique, is arguably the only available method with potential to meet all these needs. Recent developments suggest its viability for clinical application. This review addresses several of these developments and discusses remaining challenges with the emphasis on renal imaging in human subjects.

## 1. Background

The delivery of oxygen and nutrients to tissues is inextricably linked to blood flow at the level of the tissue capillary bed. This phenomenon is typically referred to as tissue perfusion and is quantified as a volume of blood delivered per unit time and mass of tissue (e.g., mL/100 g of tissue/min). In the kidneys, renal perfusion is also a key determinant of glomerular filtration. As such, the quantification of regional renal perfusion as a potential window into renal (patho) physiology has long been of interest to physiologists [1,2]. However, non-invasive, practical, and robust methods to measure renal perfusion remain elusive, particularly in the clinic [3,4]. Arterial spin labeling (ASL), a magnetic resonance imaging (MRI) technique introduced 25 years ago for quantifying tissue perfusion [5,6], is arguably the only available technique with potential to meet all these needs. Inherently non-invasive, it has been undergoing rapid developments which increasingly suggest its viability for routine clinical application. This review addresses several of these developments and discusses remaining challenges with the emphasis on renal imaging in human subjects.

### 1.1. ASL in a Nutshell

**Figure 1:**
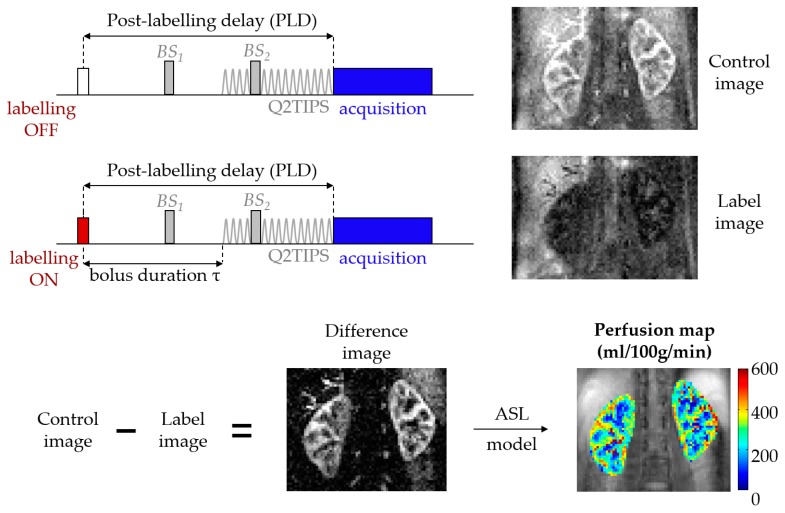
Arterial spin labeling (ASL) overview. The background suppression (BS) pulses are optional but were used to acquire the renal ASL images in this example, hence the marked difference between control and labeled images (in healthy volunteers, the amount of signal due to inflowing blood is in the order of 5% of the non-background suppressed baseline tissue magnetization). The QUIPSS II with thin-slice TI1 periodic saturation (Q2TIPS) method [7] allows one to define the bolus duration in single post-labeling delay (single-PLD) pulsed ASL (PASL) studies.

ASL is an MRI technique that harnesses blood water as a freely diffusible tracer to allow a non-invasive quantification of tissue perfusion [5,6]. In a basic ASL experiment, a label (or tag) image is flow-sensitized through the use of radiofrequency (RF) pulses that alter the longitudinal magnetization of arterial blood before it flows into the imaging plane, within the organ of interest. The acquisition is then repeated to obtain a control image, this time without perturbing the magnetization of the inflowing blood. Provided that the magnetization preparation of the inflowing blood is the only difference between the two acquisitions, simply subtracting the two resulting images yields a perfusion-weighted image (PWI). These PWIs are then fed into a model that describes the relationship between the difference signal and the actual blood perfusion. The result is a quantitative perfusion map in relevant physiological units (typically mL/100 g of tissue/min). This framework is depicted in Figure 1.

#### 1.1.1. Labeling

More than two decades of technical developments have resulted in a plethora of methods for labeling inflowing arterial blood. Interested readers are directed to more comprehensive reviews on this particular subject, such as [8,9,10]. At the time of writing this manuscript, two main approaches are used in renal ASL: (i) Pulsed ASL (PASL), particularly the Flow-sensitive Alternating Inversion Recovery (FAIR) variant; and (ii) pseudo Continuous ASL (pCASL) (see Table 1; note that less T_1_ relaxation (i.e., smaller amount of label lost during the arterial transit time) and higher labeling efficiency and Signal-to-Noise Ratio (SNR) improve ASL images). The pCASL variant has evolved from the original continuous ASL (CASL) implementations (brain: [5], kidneys: [11]). In CASL, a long-duration RF pulse is applied proximal to the region of interest while a gradient is applied in the direction of flow. This causes blood spins to be inverted as they pass through the resulting inversion plane by the process of adiabatic fast passage [12]. In pCASL, the long RF pulse used in CASL is broken down into multiple short, high-power RF pulses which can not only be optimized to reduce both magnetization transfer (MT) effects and power deposition, but are also more compatible with clinical MRI scanners. Typical B_1_ amplitudes are in the range of 1–2 µT, depending on expected flow rates and Specific Absorption Rate (SAR) constraints. On the other hand, in PASL, the inversion of a large volume of blood is achieved instantaneously (in a few milliseconds) using a frequency-modulated adiabatic inversion pulse. In the label and control conditions, Flow-sensitive Alternating Inversion Recovery (FAIR) ASL acquisitions alternate between non-spatially-selective and spatially-selective inversions, respectively. In the latter, the inversion is spatially-selective to the imaging region, so no off-resonance RF pulses are applied, making FAIR insensitive to MT effects. Regardless of the type of labeling, the ASL tracer will always decay according to the T_1_ time constant (see Section 1.1.3).

#### 1.1.2. Readout

In simple terms, ASL consists of a magnetization preparation scheme followed by a subsequent imaging module, and these two aspects of the acquisition are independent to a considerable extent. As such, ASL has been implemented with a wide variety of fast image readout techniques, which in turn can be optimized according to the specific application (see Table 2). These can be broadly classified as 2D or 3D acquisition schemes. Whereas 3D readouts are generally recommended for brain ASL [10], consensus as to which type of readout is optimal for renal applications has yet to be reached. With respect to 2D readouts, echo-planar imaging (EPI) and balanced steady-state free precession (bSSFP) sequences are frequently used in the kidneys. Optimized EPI acquisitions allow for whole-kidney coverage [14] but nevertheless provide sub-optimal and slice-dependent perfusion-weighted signal-to-noise ratios (SNR) (important drawbacks since ASL is inherently SNR-limited) and can be susceptible to off-resonance conditions. On the other hand, the short echo times and magnetization recycling in bSSFP readouts result in improved SNR and superior image quality in the presence of magnetic field inhomogeneities. However, both the increased readout duration and power deposition limit the achievable organ coverage, which is especially problematic when assessing focal disease. In recent years, 3D readouts based on the gradient and spin echo (GRASE) and rapid acquisition with relaxation enhancement (RARE) pulse sequences have been gaining traction in renal ASL (e.g., GRASE: [15]; RARE: [16]). The simultaneous excitation of the entire image volume confers several advantages to these schemes: (i) SNR efficiency; (ii) optimal background suppression; and (iii) constant post-labeling delay (PLD) across slices. The main drawback of these techniques is the image blurring caused by T_2_ decay during the data acquisition, which compromises the effective resolution of the scans. A typical approach to counteract these effects is to limit the echo train duration by acquiring *k*-space data over several segments (i.e., acquiring data over multiple excitations). While this is a relatively straightforward solution for neuroimaging applications, the increased amount and complexity of abdominal movement renders segmented acquisitions prone to image artifacts, which may severely corrupt the perfusion maps. ASL scans are typically performed at an image resolution range of 2.5–4.5 mm in plane.

#### 1.1.3. Modeling

A fundamental property of ASL is that the difference signal is inherently proportional to tissue perfusion. Nevertheless, to obtain quantitative perfusion values, several factors characterizing the ASL experiment (including labeling efficiency, label relaxation time, among others) need to be taken into account. These can be encapsulated into a kinetic model, which in the majority of ASL studies (including in the kidneys) further assumes single-compartment kinetics (see [20] for an exception in the kidneys). Even though it can be argued that such models may not accurately represent the unique anatomy and physiology of the kidneys [20], the degree to which more complex models are useful for renal ASL quantification is somewhat limited by the technical constraints currently associated with the technique (e.g., SNR, movement sensitivity).

**Figure 2:**
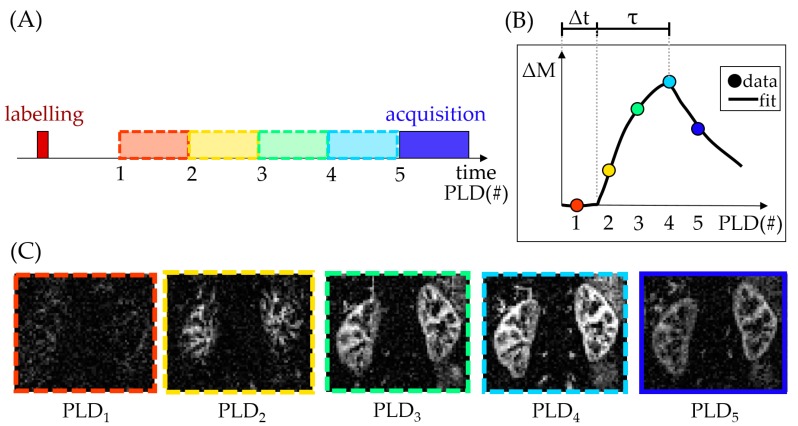
Multiple post-labeling delay (multi-PLD) ASL experiment (data acquired at a field strength of 1.5T). The schematic shows arbitrary PLDs. The actual PLDs used to acquire the ASL data in this figure were (in seconds): 0.1, 0.5, 0.9, 1.3, 2.7. (**A**) Simplified diagram of a multi-PLD acquisition. Note that after labeling, only one PLD image/volume is acquired at a time (in this case, 5 acquisitions would be necessary, each at different PLD); (**B**) Difference signal (ΔM) at each PLD and corresponding fit, highlighting parameters beyond renal blood flow (Δ*t* and *τ*); (**C**) Difference image at each PLD.

To better characterize the ASL tracer kinetics and therefore account for changes in the hemodynamic properties of tissue, the PLD (see Figure 1) can be iteratively varied in an ASL experiment (see Figure 2). The perfusion-weighted data from multiple time points across the inflow curve can then be fit, for example, to the ASL general kinetic model [21]. A benefit of this approach is that it allows calculation of parameters beyond tissue perfusion (e.g., as in [15]), such as the arterial transit time (Δ*t*) and bolus duration (*τ*) (see Figure 2). However, this comes at the cost of additional scan time, reduced perfusion-weighted SNR at each individual PLD (from a necessary reduction of the number of averages) and an increased propensity to motion artifacts.

## 2. Recent Developments in Renal ASL

**Figure 3:**
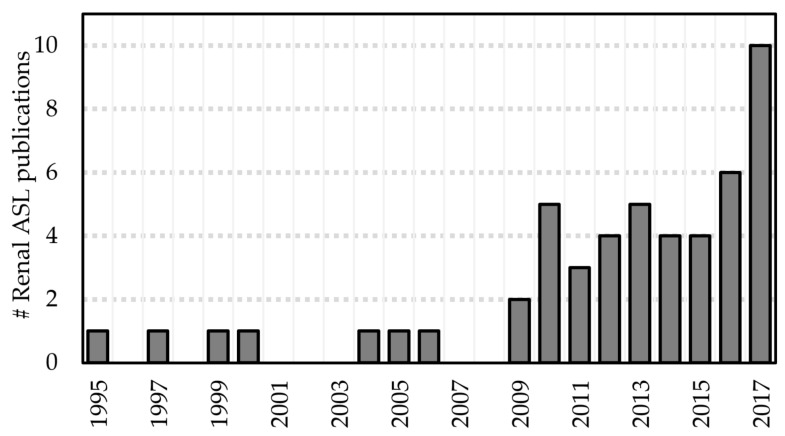
Number of renal ASL publications in humans per year since the introduction of the technique (excluding cancer studies and conference proceedings) [11,14,15,16,17,18,19,20,22,23,24,25,26,27,28,29,30,31,32,33,34,35,36,37,38,39,40,41,42,43,44,45,46,47,48,49,50,51,52,53,54,55,56,57,58,59,60,61,62,63] (*n* = 50).

Renal ASL has recently become a more active area of research, with approximately 30 studies published in the last five years (see Figure 3). Commensurate developments have been described in the literature. For the purposes of this review, these developments can be categorized as methodological developments and emerging clinical applications.

### 2.1. Methodological Developments

#### 2.1.1. Validation of ASL Renal Blood Flow Measurements

Initial validation studies are crucial for the establishment of emerging techniques. Renal ASL has been compared to alternative methods to assess renal blood flow (RBF), such as the gold standard method of para-aminohippurate clearance [28], microspheres [64], ultrasound [65], scintigraphy [17], and contrast-enhanced MRI [32,46,59,66,67]. These generally support the hypothesis that ASL can provide realistic estimates of RBF. Nevertheless, it is worth noting that with respect to the comparisons with dynamic contrast-enhanced MRI (DCE-MRI), there is conflicting evidence with respect to the similarity of RBF estimates obtained with the two techniques (see Table 3).

This suggests that there are methodological differences (e.g., different kinetic properties of the tracers used in each of the techniques due to the size difference between the tracer molecules as well as relaxation effects of the ASL tracer) still to be resolved between the techniques, a task which is complicated by the lack of a clinical gold standard for RBF measurements. The latter point motivates another class of ASL validation studies that aim to characterize the reproducibility of the technique. Several groups have shown good repeatability of cortical RBF in healthy volunteers [15,44,52,60] and transplant patients [30,34], while medulla measurements are less robust [32,34,60]. RBF estimates obtained from ASL have been shown to be more reproducible than those obtained from DCE-MRI [46].

#### 2.1.2. Improving the Robustness of RBF Measurements

Several renal ASL studies employing multi-PLD acquisitions have been published in the last five years [15,17,19,39,46,50,57,59,60]. The main features of these studies are summarized in Table 4. As detailed in Section 1.1.3, parameters in addition to RBF can be obtained: arterial transit time (Δ*t*) and bolus width (*τ*). These may be important clinical variables on their own, as shown in neuroimaging [68,69]. This is likely to hold true in certain renal pathologies (e.g., renal stenosis). Some of the variation in arterial transit time (Δ*t*) between different studies is likely to be caused by different labeling schemes. This may occur when different labeling techniques result in a different position for the leading edge of the bolus, as would be the case when using PASL (FAIR) (e.g., [15,19]) or pCASL (e.g., [17,60]). Data regarding quantification of these parameters in the kidneys remains relatively sparse. This may be due to the focus on removing the effect of these parameters as confounds in the main RBF quantification, rather than of quantification on these parameters themselves [39]. In fact, a possibility presented by Dai et al. [70] is to perform a quick, low-resolution multi-PLD scan for transit time mapping to optimize a subsequent high-resolution single-PLD ASL scan.

#### 2.1.3. ASL at High Field

Ultra-high field imaging provides an inherent increase in SNR, which in ASL is further enhanced by an increase in label T_1_ relaxation times. This results in a reduced loss of label en route to the tissue of interest. This is offset by technical challenges such as increased B_0_ and B_1_ field inhomogeneity (which affects image quality) and power deposition (which limits organ coverage). Nevertheless, by carefully addressing these challenges, Li et al. have recently shown renal ASL to be feasible at 7T [19].

### 2.2. Clinical Applications

ASL is able to detect perfusion differences between healthy and diseased kidneys, both in chronic kidney disease (CKD) [35,45,55,57] and acute kidney injury (AKI) [41]. For example, recent studies have shown that ASL was able to detect significantly reduced RBF in diabetic patients compared to healthy volunteers [58,61], as well as small changes in RBF across CKD stages [58]. Renal ASL has also been applied to patients with metabolic syndrome [28] and chronic heart failure [49]. Several studies showed the usefulness of ASL for the assessment of renal cell carcinoma [16,71,72,73,74,75]. Two recent studies have shown decreased renal blood flow in older (>40 years of age) compared to younger adult subjects (<40 years of age) [17,55]. This is consistent with an expected age-associated reduction of renal function [76].

#### 2.2.1. Monitoring Renal Allograft Function

ASL has been used to assess RBF in renal transplantation in multiple studies [30,33,43,48,50,51,53]. ASL RBF has been shown to correlate to estimated glomerular filtration rate (eGFR) [43,48,51], and provided clinically relevant information (e.g., in [48] transplant patients with delayed graft function were shown to have significantly reduced perfusion). Two longitudinal studies have shown the potential of ASL for monitoring RBF in both transplant donors and recipients [50,53].

#### 2.2.2. Pharmacological Modulation

The effects of a variety of drugs on renal perfusion have been assessed with ASL. For example, RBF has been shown to increase during aliskiren therapy and return to original levels after withdrawal of the drug [36]. Others include telmisartan [28], furosemide [37], losartan [53] and captopril [54]. The effects of renal denervation (RDN) on RBF in a group of patients with treatment-resistant hypertension were also assessed with ASL (no changes detected up to 3 months after RDN) [38]. Chowdhury et al. assessed changes in cortical RBF in healthy volunteers following administration of colloid fluids [77].

## 3. Challenges

### 3.1. Subject Movement

Subject movement poses a significant challenge as it can corrupt ASL data at multiple stages. Errors in the perfusion-weighted data arise from the ASL subtraction step if the position of the kidneys in the control and label data is inconsistent (e.g., due to movement). Furthermore, ASL acquisitions require multiple image volumes to be acquired (either for the purposes of signal averaging or multi-PLD sampling). High-quality perfusion maps can only be obtained if there are techniques in place to ensure that not only individual images are artifact-free, but also that the kidneys are in a consistent position throughout the time series. Several techniques are available to address patient movement (see Table 5), many of which can be used in combination.

Breath-hold ASL acquisitions (e.g., [17,19,35,59]) are generally long and certain patient populations are not capable of complying to the high number of breath-holds necessary [59,78], or breath-holds of longer duration [37,41]. Synchronized breathing (e.g., [16,33]) arguably requires even higher levels of patient compliance. Respiratory-triggering using bellows allows scans to be performed under free-breathing [15,79], but lengthens the scan time and does not compensate for respiratory movement completely. Standard respiratory triggering implementations may require modifications (e.g., [55]) to cope with the time gap between trigger and data acquisition, particularly in multi-PLD protocols. Alternatively, MRI navigators that track the lung/liver interface can be used for prospective motion correction [29,45,56].

Even though background-suppression is recommended for physiological motion mitigation in brain ASL [10], more research is needed to ascertain the applicability of this recommendation for renal ASL. Preliminary work has provided inconclusive data with regards to this question [14,27]. Importantly, strong background-suppression may decrease the effectiveness of image registration algorithms [10,46]. Signal averaging (used in the majority of renal ASL studies acquiring up to 100 control/label pairs [61], even though acquisitions with a range of 20–30 pairs are more common) suppresses artifacts, boosts perfusion-weighted SNR, and enables data rejection approaches (e.g., [27]) in the post-processing stage at the expense of additional scan time. Rejection of corrupted data can be performed by visual inspection of the data (e.g., [79]), or using automatic approaches, such as [58,80,81,82]. Image registration is particularly advantageous as it preserves SNR. Importantly, because the kidneys move independently ([83,84]), both should be masked for independent registration ([26,33]) unless non-rigid transformations are used (e.g., [41,58]). Interestingly, studies involving transplant patients may be less affected by breathing-related movement due to the location of the kidney allograft [30,51].

### 3.2. Lack of Consensus Regarding Labeling Strategy

At the time of writing, no direct comparison between PASL and pCASL in renal ASL has been performed. Furthermore, optimal labeling parameters for each approach remain unknown. For example, single-PLD FAIR renal ASL studies have used PLDs ranging from 0.9 s [44] to 2.0 s [61] (the latter in CKD patients). Whereas pCASL is widely accepted as the optimal labeling approach in brain ASL [10], labeling efficiency in the aorta may be reduced due to a combination of susceptibility effects and pulsatile flow [17]. Multi-PLD studies (e.g., [15,17,59] can be more robust to changes in kidney hemodynamics, such as in the case of delayed arrival times (which can be misinterpreted as low RBF in single-PLD studies) and have the potential to provide clinically-relevant information beyond RBF (see Figure 2), but are technically demanding (lower SNR and greater propensity for motion artifacts).

### 3.3. Readout Optimization

The majority of early renal ASL studies focused on single-slice acquisitions. More recently, accelerated 2D multi-slice [14] and volumetric acquisitions [15,16,57] allow whole kidney coverage. This will enhance the diagnostic capabilities of ASL, especially in the assessment of focal disease (e.g., [16]). However, even though some studies acquire multi-slice datasets, often regions-of-interest are only drawn in a limited number of slices [15,57,60], under the assumption of homogeneous perfusion deficits in CKD [57]. As such, the clinical need may dictate the optimal ASL readout: whereas volumetric acquisitions may provide whole kidney coverage at the expense of lower resolution and/or increased motion sensitivity, single-slice methods may provide higher resolution data, which will reduce partial volume effects, therefore enhancing cortico-medullary differentiation.

### 3.4. Lack of Consensus Regarding Analysis Approach

#### 3.4.1. Quantification Model Selection

As detailed in Section 1.1.3, the majority of renal ASL studies employ single-compartment models. Nevertheless, these can be built on different assumptions [85]. Consequently, the RBF values obtained from a given dataset may differ according to the chosen model. Furthermore, the RBF changes in the presence of pathology may also depend on the model assumptions. For example, if assuming short arrival times and instantaneous exchange of blood water with tissue water, the decay of the label is governed by the T_1_ of tissue. Alternatively, if negligible exchange is assumed (i.e., label remains in the capillary bed before it decays), then the T_1_ of blood describes the label decay. Some studies have assumed the T_1_ of blood and renal cortex to be equivalent (e.g., [61]), where others account for the decay of the label initially in the vasculature and then after exchanging with tissue [15,17,55]. Furthermore, when utilizing a model which considers the T_1_ of tissue, it is possible to use a literature T_1_ (e.g., [16,17,53]), or measure T_1_ with a separate acquisition in the same scanning session (e.g., [15,55]). The former shortens the acquisition protocol and is less prone to introduce artifacts in the RBF maps (from residual misalignment between T_1_ mapping data and ASL data). However, measuring T_1_ on a subject basis may be especially advantageous when scanning patient cohorts as tissue T_1_ may change (increase, e.g., due to fibrosis or inflammation) with greater levels of renal function impairment [49,62,86].

#### 3.4.2. Region of Interest (ROI) Definition

RBF measurements are commonly reported as the mean RBF across ROIs, which are manually segmented in the majority of cases (exceptions e.g., [49,52,55]). Some studies define ROIs for perfusion analysis based on M_0_ (i.e., “anatomical”) data [37,57], while others use the perfusion-weighted data [16,19] or even the calculated RBF maps directly for ROI definition [58,61,62], making the resulting measures somewhat operator-dependent. Additionally, in patient groups with focal perfusion deficits, the ASL signal will be markedly reduced in affected areas. If the PWIs are used for ROI drawing, this may lead to biases, such as failing to include anatomically identifiable cortex because reduced perfusion causes it to be undetectable in the PWIs. Factors which further complicate ROI definition include the relatively low spatial resolution necessary for ASL measurements [56] as well as movement artifacts [16].

## 4. Conclusions

ASL is, uniquely, a completely non-invasive, broadly applicable quantitative tissue perfusion mapping MRI technique. Renal ASL has been shown to be technically feasible and potential clinical applications have been demonstrated in small, single-centre patient studies. ASL is poised to be an important component of multi-parametric renal MRI studies in the future, as recently demonstrated (e.g., [55,63]). Standardization and streamlining of image acquisition, processing and analysis methods (e.g., real-time calculation of ASL parameter maps) as well as multi-centre studies will be crucial for wider uptake and evaluating the utility of renal ASL in the clinic and its impact for patient management.

## Figures and Tables

**Table 1:** General comparison of labeling schemes in ASL.

Technique	Labeling	Temporal Bolus Width	T_1_ Relaxation	Label Efficiency	SNR	Robustness
PASL (FAIR)	Spatial	Unknown *	More	More	Less	More
pCASL	Temporal	Labeling duration	Less	Less	More	Less

* Dependent on coil coverage and anatomy, however state-of-the-art Flow-sensitive Alternating Inversion Recovery (FAIR) implementations frequently use Quantitative imaging of perfusion using a single subtraction II (QUIPSS II) [13]/Q2TIPS [7] methods to define the temporal bolus width. SNR: Signal-to-Noise Ratio.

**Table 2:** General comparison of image readout schemes in ASL.

Readout	Nominal SNR	Spatial Resolution	Robustness to Motion	Background Suppression	Post-Labeling Delay	Typical Sequences
2D (single or multislice)	✓	✓✓✓	✓✓	Slice-dependent	Slice-dependent	EPI [17], bSSFP [18]
3D (segmented)	✓✓✓	✓✓	✓	Strongest, constant across slices	Constant across slices	GRASE [15], RARE [19]
3D (single-shot)	✓✓	✓	✓✓✓			

Checkmarks mean better for each “feature” of the readout type (e.g., 3D single-shot is most robust concerning motion artifacts, but achieves the lowest spatial resolution, due to echo train duration constraints).

**Table 3:** Comparison of RBF estimates obtained by ASL and dynamic contrast-enhanced MRI (DCE-MRI).

Reference	*n*	RBF (mL/100/min) *		*p*-Value (*t*-Test)
		ASL	DCE	
Winter et al. [66]	6 rabbits	328 ± 59	298 ± 60	>0.05
Wu et al. [32]	19 humans	227 ± 30	272 ± 60	<0.001
Zimmer et al. [67]	6 rats	HK: 416 ± 124	HK: 542 ± 85	<0.01
		AKI: 316 ± 102	AKI: 407 ± 119	<0.01
Cutajar et al. [46]	16 humans	263 ± 41	287 ± 70	0.43
Conlin et al. [59]	7 humans	151 ± 37 mL/min	152 ± 41 mL/min	N/A

* Except in [59]; HK: healthy kidney; AKI: acute kidney injury (contralateral).

**Table 4:** Overview of renal ASL studies using multiple post-labeling delays.

Reference	Labeling	PLD (s) (n)	Multi-PLD Fit	Mean RBF (mL/100 g/min) *	Δt *	Τ *	Quantification Highlights
[15]	FAIR	0.1:0.2:2.7 (14)	Yes	196 and 204 (two scans)	143 ± 45 ms	N/A	1st multi-PLD study. Repeatable ASL parameters.
[39]	EPISTAR	0.25:0.1:1.85 (17)	No	287 ± 49	N/A	N/A	Single-PLD quantification at highest signal PLD (peak time = 1330 ± 148 ms).
[46]	FAIR	0.1:0.2:2.7 (14)	Yes	263 ± 41	0.3 ± 0.7 s	1.2 ± 0.2	ASL and DCE agree. ASL more repeatable.
[50]	FAIR	0.1:0.2:2.7 (14)	Yes	Pre/post-nephrectomy: 186 ± 36/184 ± 37	N/A	N/A	First study to assess RBF in healthy living kidney donors, pre and post-donation.
[17]	pCASL	0.5:0.5:1.5 (3)	Yes	Young/older: 157 ± 38/117 ± 24	Young/older (ms): 961 ± 260/1228 ± 227	pCASL-defined (2.0)	Higher RBF/shorter Δt in young subjects.
[19]	FAIR	0.3:0.3:2.1 (7)	Yes	309 ± 31	110 ± 26 ms	702 ± 69 ms	RBF from multi-PLD and single-PLD study similar.
[57]	FAIR	1.2:0.2:2 (5)	No	Healthy subjects/Patients: 191 ± 9/102 ± 11 at PLD = 1.8 s	700 ms (assumed)	N/A	RBF increased at higher PLDs.
[59]	FAIR	0.15 + 0.2:0.1:1.6 (16)	Yes	Healthy subjects/Patients (mL/min): 151 ± 37/158 ± 103	N/A	N/A	RBF derived from slope of ASL difference signal.
[60]	pCASL	0.5:0.5:2.0 (4)	Yes	215 ± 65	1141 ± 262 ms	pCASL-defined (2.0)	Cortical RBF repeatable. Poor reproducibility of cortical Δt, medullary RBF/Δt.

* Values only shown for the renal cortex.

**Table 5:** Motion correction strategies most relevant for renal ASL.

Motion Correction Technique		Prospective	Retrospective	Extra Setup Time	Extra Scan Time	Patient-Friendly	Easily Available	Time-Consuming Post-Processing
Breath-holding	Traditional	✓	✗	✓	✓	✗	✓	✗
	Synchronized breathing	✓	✗	✓	✓	✗	✓	✗
Respiratory-triggering (bellows)		✓	✗	✓	✓	✓	✓	✗
MR-navigators		✓	✗	✗	✓	✓	✗	✗
Snapshot Imaging		✓	✗	✗	✗	✓	✓	✗
Background-suppression		✓	✗	✗	✗	✓	✗	✗
Signal averaging		✓	✓	✗	✓	✗	✓	✗
Data rejection	Visual sorting	✗	✓	✗	✗	✓	✓	✓
	Automatic approaches	✗	✓	✗	✗	✓	✗	✗
Image registration		✗	✓	✗	✗	✓	✓	✓

Check mark: Yes; ✗: No.
